# Age and Treatment Efficacy in Myasthenia Gravis: A Systematic Review, Meta‐Analysis, and Meta‐Regression

**DOI:** 10.1111/ene.70737

**Published:** 2026-08-12

**Authors:** Christopher Nelke, Marion Skrobol, Lukas Theissen, Francesco Sacca, Tobias Ruck

**Affiliations:** ^1^ Department of Neurology, BG University Hospital Bergmannsheil Ruhr University Bochum Bochum Germany; ^2^ BG University Hospital Bergmannsheil Heimer Institute for Muscle Research Bochum Germany; ^3^ Department of Neurology, Medical Faculty Heinrich Heine University Duesseldorf Duesseldorf Germany; ^4^ GENESIS Department Federico II University of Naples Naples Italy

**Keywords:** age, complement inhibitor, FcRn inhibitor, meta regression, meta‐analysis, myasthenia gravis

## Abstract

**Background:**

Myasthenia gravis (MG) is increasingly concentrated in older adults, and age may shift the balance of benefit and risk for MG therapies. This study seeks to evaluate the effect of age on treatment efficacy in clinical trials of MG.

**Methods:**

PubMed and Cochrane Library were searched and clinical trials enrolling adults with MG that reported the quantitative myasthenia gravis (QMG) score and the myasthenia gravis activities of daily living (MG‐ADL) scale were included. Of 2214 records identified, 18 trials met inclusion criteria. Random‐effects meta‐analysis was used to pool treatment effects, and random‐effects meta‐regression evaluated trial‐level mean baseline age as a modifier of treatment efficacy.

**Results:**

Eighteen trials published from 2017 to 2025 included 1823 participants (982 assigned to treatment and 841 to control). Pooled effects favored treatment over control for the QMG (MD −2.402; 95% CI, −3.099 to −1.706; $I^{2}$ = 60.7%) and MG‐ADL scores (MD, −1.591; 95% CI, −1.874 to −1.309; $I^{2}$ = 0.0%). In meta‐regression, higher mean trial age was associated with attenuation of treatment benefit on the QMG scale (*β* per 10 years, 1.21; 95% CI, 0.02 to 2.42; *p* = 0.047). This effect was most pronounced for FcRn inhibitors. No association was observed for the MG‐ADL score (*β* per 10 years, 0.08; 95% CI, −0.50 to 0.66; *p* = 0.779).

**Conclusions:**

In this meta‐analysis, older trial populations showed smaller treatment‐related improvements in the QMG but not in the MG‐ADL score. These findings suggest that age may contribute to differences in treatment effects across MG trials.

## Graphical abstract

Across 18 randomized trials involving 1823 participants, active therapies improved both QMG and MG‐ADL outcomes versus control. Trial‐level meta‐regression showed that each 10‐year increase in mean baseline age was associated with a 1.21‐point smaller QMG benefit, explaining 25.4% of between‐trial heterogeneity; the association appeared strongest in FcRn inhibitor trials. No age association was observed for MG‐ADL, supporting patient‐level studies of age‐related differences in treatment response.

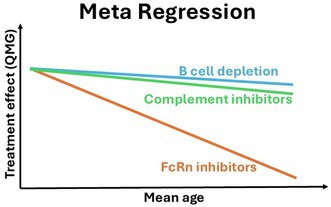

## 1 Introduction

Myasthenia gravis (MG) is an antibody‐mediated disease affecting neuromuscular transmission [1, 2, 3]. Over the past decade, MG therapeutics have expanded beyond broad immunosuppression to include mechanism‐targeted agents, including inhibitors of the neonatal Fc receptor (FcRn) [4, 5, 6, 7, 8] and the complement pathway (C5IT) [9, 10, 11, 12]. With multiple strategies advancing, the field is increasingly focused not only on overall efficacy but on whether treatment effects vary meaningfully across patient subgroups.

In parallel, the burden of MG is increasingly concentrated in older adults, reflecting population aging and an evolving epidemiology [13, 14]. Older patients commonly have higher comorbidity burden and differ in immune function and pharmacologic vulnerability—factors that influence both the benefit and risk of targeted immunotherapies [15, 16]. However, clinical trials are often designed to estimate average treatment effects; age‐stratified efficacy estimates are variably reported and have not been consistently synthesized across therapeutic mechanisms.

To target this knowledge gap, we conducted a meta‐analysis and meta‐regression of randomized clinical trials of MG therapeutics to evaluate baseline age as a modifier of treatment efficacy. Treatment effects were summarized as mean differences in change from baseline on the quantitative myasthenia gravis (QMG) and the myasthenia gravis activities of daily living (MG‐ADL) scales, with subgroup analyses to contextualize findings across FcRn‐, C5‐ and B cell‐targeted interventions.

## 2 Methods

### 2.1 Study Design and Reporting

This study was designed as a systematic review with random‐effects meta‐analysis and prespecified meta‐regression to evaluate age as an effect modifier of the treatment response in randomized clinical trials of MG. Reporting follows PRISMA 2020 recommendations (Figure S1). The protocol was preregistered in Prospero as CRD420251152544. Because this work synthesizes published, aggregate‐level data, institutional review board approval and informed consent were not required.

### 2.2 Data Sources and Searches

We systematically searched MEDLINE via PubMed and the Cochrane Library from inception to 03/2026. The search strategy combined terms for myasthenia gravis and interventional treatment studies. Baseline age was extracted from eligible trials and was not required as a search term for study search. We applied the truncation (*) to cover variable spellings. Search terms within each key domain were combined using “OR”. These were then linked using “AND” to establish a logical connection between the four search components. To ensure no eligible study was missed from our search, the references of the included articles were manually screened.

### 2.3 Study Selection and Data Extraction

Eligible studies were randomized, controlled clinical trials for pharmacological interventions enrolling adult patients with MG that compared active treatment with placebo/control and reported sufficient data to estimate a treatment effect on at least one prespecified (QMG or MG‐ADL). Key inclusion and exclusion criteria are presented in Table S1. Two reviewers (CN and MS) independently screened titles/abstracts and full texts. Disagreements were resolved by consensus or a third reviewer (TR). Reasons for exclusion at the full‐text stage were documented. Duplicates were removed using EndNote's duplicate detection feature (which compares title, author and publication year), followed by manual verification.

Two reviewers (CN and MS) independently extracted data using a piloted form. Extracted variables included trial design, sample sizes, intervention details, baseline mean age (and dispersion), and outcome data (MG‐ADL and QMG). Any discrepancies were resolved by consensus.

### 2.4 Multi‐Arm Trials With a Shared Placebo

For trials with multiple treatment arms and a single placebo group, we avoided double‐counting the shared placebo. We used one of two prespecified approaches depending on available data.

#### 2.4.1 Contrast‐level pooling

If adjusted contrasts and their standard error (SE) or confidence interval (CI) were available for each active arm, we pooled contrasts using inverse‐variance methods:

$${\hat{\theta }}_{\text{pooled}}=\frac{\sum w_{j}{\hat{\theta }}_{j}}{\sum w_{j}},w_{j}=\frac{1}{{\mathrm{SE}}_{j}^{2}},{\mathrm{SE}}_{\text{pooled}}=\sqrt{\frac{1}{\sum w_{j}}}$$

Because correlations between adjusted contrasts sharing a common placebo are not typically reported, this pooling assumes independence between arm‐specific contrasts.

If the mean and SD were available for each treatment group, we combined these into a single pooled treatment group using the continuous‐outcome formula as recommended by the Cochrane handbook:

$${\overset{`}{x}}_{\text{pooled}}=\frac{\sum n_{j}{\overset{`}{x}}_{j}}{\sum n_{j}}$$

$${\mathrm{SD}}_{\text{pooled}}=\sqrt{\frac{\sum \left(n_{j}-1\right){\mathrm{SD}}_{j}^{2}+\sum n_{j}{\left({\overset{`}{x}}_{j}-{\overset{`}{x}}_{\text{pooled}}\right)}^{2}}{\left(\sum n_{j}\right)-1}}$$

and then computed MD and SE (MD) versus the single placebo group.

### 2.5 Outcomes and Effect Measure

The prespecified effect measure was the mean difference (MD) in change from baseline between treatment and control/placebo for the continuous outcomes QMG and MG‐ADL scores; both assessed at each trial's prespecified primary efficacy time point. For each included study, we extracted, or derived as detailed below, the study‐level estimate $y_{i}$(MD in change from baseline) and its standard error ${\mathrm{SE}}_{i}$. Negative MD values indicate greater improvement with treatment (larger reductions in QMG/MG‐ADL).

### 2.6 Derivation of Study‐Level Estimates and Standard Errors

For each trial, we preferentially used the placebo‐adjusted model‐based treatment contrast (e.g., least‐squares mean difference) when reported, reflecting the prespecified primary estimand. When a placebo‐adjusted contrast was accompanied by a confidence interval (CI), we derived the standard error (SE) using the normal approximation:

$${\mathrm{SE}}_{i}=\frac{\mathrm{UCL}-\mathrm{LCL}}{2\;z_{1-\alpha /2}}$$

with $z_{0.975}=1.96$ for a 95% CI; for a 90% interval, $z_{0.95}=1.645$. SEs were derived from reported CIs using a normal approximation when degrees of freedom were not reported.

If a trial reported group‐level mean and standard deviation (SD) by arm rather than a direct adjusted contrast, we calculated:

$$\mathrm{MD}={\overset{`}{x}}_{T}-{\overset{`}{x}}_{C},\mathrm{SE}\left(\mathrm{MD}\right)=\sqrt{\frac{{\mathrm{SD}}_{T}^{2}}{n_{T}}+\frac{{\mathrm{SD}}_{C}^{2}}{n_{C}}}$$

If a trial reported standard errors (SE) for arm‐level means rather than SDs, we converted using:

$$\mathrm{SD}=\mathrm{SE}\times \sqrt{n}$$

and then computed SE (MD) as above.

### 2.7 Random‐Effects Meta‐Analysis and Meta‐Regression

We assessed whether trial‐level mean baseline age was associated with treatment effects using random‐effects meta‐regression, conducted separately for QMG and MG‐ADL:

$$y_{i}=\beta_{0}+\beta_{1}{\mathrm{Age}}_{i}+u_{i}+\varepsilon_{i},\text{with}\;u_{i}∼N\left(0,\tau^{2}\right)\;\text{and}\;\varepsilon_{i}∼N\left(0,{\mathrm{SE}}_{i}^{2}\right).$$

To improve interpretability, age was scaled per 10 years and mean‐centered within each analysis set; β is reported as the change in mean difference (MD; treatment –control) per 10‐year increase in mean trial age. Inference used Knapp–Hartung (Hartung–Knapp–Sidik–Jonkman) small‐sample adjustments [17]. To evaluate whether age modification differed by mechanism, we repeated meta‐regression within prespecified treatment classes (FcRn inhibitors, C5IT and B cell‐targeted therapies), with age re‐centered within each subset. We report residual between‐study variance from the meta‐regression ($\tau_{\text{residual}}^{2}$), the corresponding residual $I^{2}$, and the proportion of heterogeneity explained by age ($R^{2}=\left(\tau^{2}-\tau_{\text{residual}}^{2}\right)/\tau^{2}$). Bubble plots display study effects versus mean age with fitted meta‐regression lines and 95% confidence bands; bubble size is proportional to the random‐effects inverse‐variance weight $w_{i}=1/\left({\mathrm{SE}}_{i}^{2}+\tau^{2}\right)$. For interpretability, the *y*‐axis is inverted.

### 2.8 Sensitivity Analyses

We conducted sensitivity analyses to evaluate the robustness of the age meta‐regression findings for QMG and MG‐ADL. For each sensitivity set, we refit random‐effects meta‐regression models with the study‐level MD in change from baseline (treatment –control) as the dependent variable and mean trial age as a continuous moderator, centered within each analysis set and scaled per 10‐year increase. Models used inverse‐variance weighting with REML estimation of between‐study variance ($\tau^{2}$) and Knapp–Hartung–type small‐sample inference for moderator confidence intervals. Sensitivity sets included excluding inputs based on median‐imputed summaries, restricting to effect estimates derived from reported placebo‐adjusted contrasts versus those derived from arm‐level group SDs, and restricting the analysis to targeted therapeutic classes.

### 2.9 Risk of Bias Assessment

Risk of bias for each trial and outcome was assessed using the Cochrane Risk of Bias 2 tool. Two reviewers (CN and MS) assessed risk of bias independently with discrepancies resolved by consensus. No studies were excluded due to high risk of bias.

### 2.10 Software and Reproducibility

Analyses were conducted in R (v4.5.3) using the meta package (v8.2 with metagen for random‐effects meta‐analysis and metareg for meta‐regression) with REML heterogeneity estimation and Hartung–Knapp inference.

## 3 Results

### 3.1 Study Selection and Characteristics

Overall, 18 randomized clinical trials published between 2017 and 2025 met the inclusion criteria, comprising 1823 participants (982 assigned to treatment and 841 to control) (Table 1).

**TABLE 1:** Included studies and inputs.

Study	Year	Treatment class	Agent	*N* treatment/*N* control	MG‐ADL MD (SE)	QMG MD (SE)	Mean age (years)	Time after baseline used for analysis (weeks)
Howard et al. 2017	2017	C5IT	Eculizumab	62/63	−1.80 (0.68)	−2.90 (0.85)	47.2	26
Zhou, L. et al. 2017	2017	SIT	Tacrolimus	44/38	−0.90 (0.57)	−1.70 (0.92)	42.4	24
Hewett, K. et al. 2018	2018	BAFF inhibitor	Belimumab	18/21	−0.31 (0.84)	−1.84 (0.36)	56.1	24
Howard, J. F., Jr. et al. 2019	2019	FCRN	Efgartigimod	12/12	−2.05 (0.96)	−2.38 (1.14)	49.4	4.14*
Howard, J. F. et al. 2020	2020	C5IT	Zilucoplan	29/15	−2.25 (1.20)	−2.55 (0.91)	49.6	12
Bril, V. et al. 2021	2021	FCRN	Rozanolixizumab	21/22	−1.40 (0.61)	−0.70 (0.91)	51.9	4.14
Nowak, R. J. et al. 2022	2022	CD20 mAb	Rituximab	25/27	−0.70 (0.90)	−2.30 (1.32)	55.1	52
Piehl, F. et al. 2022	2022	CD20 mAb	Rituximab	25/22	−1.20 (0.92)	−1.10 (1.49)	63.0	26 (QMG) 16 (ADL)
Vu et al. 2022	2022	C5IT	Ravulizumab	86/89	−1.60 (0.53)	−2.00 (0.62)	55.6	26
Yan, C. et al. 2022	2022	FCRN	Batoclimab	21/9	−2.32 (1.07)	−6.70 (1.44)	39.1	6.14
Bril et al. 2023	2023	FCRN	Rozanolixizumab	133/67	−2.61 (0.51)	−4.13 (0.72)	50.4	6.14
Howard et al. 2023	2023	C5IT	Zilucoplan	86/88	−2.09 (0.58)	−2.94 (0.73)	53.0	12
Antozzi et al. 2024	2024	FCRN	Nipocalimab	54/14	−1.16 (0.96)	−0.09 (0.97)	57.4	8.14
GomezMancilla, B. et al. 2023	2024	*B* cell‐targeted (hCD40 mAb)	Iscalimab	17/17	−1.45 (1.02)	−1.14 (1.38)	44.0	25
Yan et al. 2024	2024	FCRN	Batoclimab	67/64	−1.90 (0.46)	−4.70 (0.69)	43.8	6^a^
Antozzi et al. 2025	2025	FCRN	Nipocalimab	77/76	−1.45 (0.47)	−2.81 (0.71)	52.4	22 to 24^b^
Nowak et al. 2025	2025	*B* cell‐targeted	Inebilizumab	119/117	−1.90 (0.49)	−2.50 (0.66)	47.5	26
Habib et al. 2025	2025	IL‐6 inhibitor	Satralizumab	86/80	−1.02 (0.44)	−1.63 (0.59)	47	24

Abbreviations: C5IT, complement inhibitor; FCRN, FcRn inhibitors; mAB, monoclonal antibody; MD, mean difference (treatment –placebo) in change from baseline; MG‐ADL, myasthenia gravis activities of daily living; QMG, quantitative myasthenia gravis; SE, standard error; SIT, standard immunosuppressant.

^a^ Time of maximum difference as reported in the study.

^b^ Mean change of weeks 22, 23 and 24 as reported in the study.

The included trials evaluated multiple therapeutic mechanisms, most commonly FcRn inhibitors (7 trials; 643 participants), C5IT (4 trials; 518 participants) and *B* cell‐targeted therapies (4 trials; 372 participants). Additional studies evaluated BAFF inhibition with belimumab, IL‐6 inhibition with satralizumab, and standard immunosuppression (SIT) with tacrolimus. Trial‐level mean baseline age ranged from 39.1 to 63.0 years (mean [SD], 50.2 [6.0] years). All included trials contributed effect estimates for both prespecified outcomes as change from baseline.

### 3.2 Risk of Bias

Overall risk of bias was low for most domains across the included trials (Figure S2). Most frequently, concerns were identified for missing outcome data and selection of the reported result, whereas domains related to the randomization process, deviations from intended interventions, and outcome measurement were predominantly judged as low risk of bias.

### 3.3 Primary Meta‐Analysis and Pooled Effects

**FIGURE 1:**
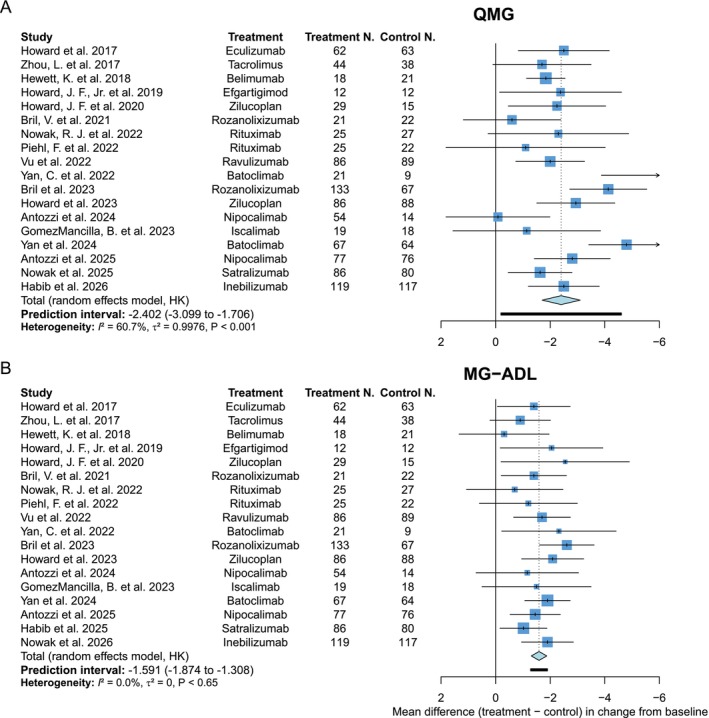
Meta‐analysis of treatment effects in myasthenia gravis. (A) Quantitative myasthenia gravis (QMG) score. (B) Myasthenia gravis activities of daily living (MG‐ADL) scale. For each trial, squares indicate study‐specific mean differences (treatment –control) in change from baseline at the prespecified primary efficacy time point, with square size proportional to the study weight; horizontal lines indicate 95% CIs. A diamond indicates the pooled random‐effects estimates (Hartung–Knapp method); whiskers below the pooled estimate indicate the 95% prediction interval. The solid vertical line denotes no between‐group difference; negative values favor treatment. CI, confidence interval; HK, Hartung–Knapp; MD, mean difference; MG, myasthenia gravis.

Across 18 trials including 1823 participants, treatment was associated with greater improvement in QMG compared with control (pooled MD, −2.402; 95% CI, −3.099 to −1.706; $I^{2}$ = 60.7%; $\tau^{2}$ = 0.998) (Figure 1A). Negative MD values indicate greater improvement. Treatment was similarly associated with greater improvement in MG‐ADL compared with control (pooled MD, −1.591; 95% CI, −1.874 to −1.309; $I^{2}$ = 0.0%; $\tau^{2}$ = 0.000) (Figure 1B). Heterogeneity was considerable for QMG but negligible for MG‐ADL, which may reflect greater between‐trial variability in examiner‐assessed impairment than in patient‐reported scores.

### 3.4 Meta‐Regression With Age

#### 3.4.1 QMG

**FIGURE 2:**
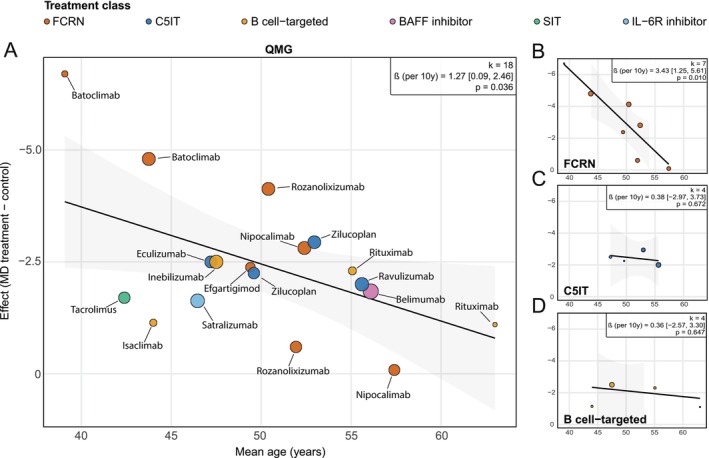
Association of age with the treatment effect on the QMG score. (A) Bubble plot of trial‐specific mean differences (treatment –control) in change from baseline for the quantitative myasthenia gravis (QMG) score, plotted against mean baseline age. Bubble size is proportional to the inverse‐variance weight in the random‐effects model. The solid line indicates the fitted meta‐regression; shading indicates the 95% confidence interval (CI) around the fitted line. The *y*‐axis is inverted so that more negative values (greater improvement with treatment) appear higher. (B) Meta‐regression restricted to FcRn inhibitor trials. (C) Meta‐regression restricted to complement inhibitor (C5IT) trials. (D) Meta‐regression restricted to *B* cell‐targeting trials. Treatment classes are indicated by point color. BAFF, *B*‐cell activating factor; CI, confidence interval; C5IT, complement C5 inhibitor therapy; CD20‐mAb, anti‐CD20 monoclonal antibody; FcRn, neonatal Fc receptor; MD, mean difference; MG, myasthenia gravis; nCD40 mAb, anti‐CD40 monoclonal antibody; QMG, quantitative myasthenia gravis; SIT, standard immunosuppressive therapy.

In random‐effects meta‐regression, higher trial‐level mean age was associated with attenuation of the treatment effect on the QMG score (*β* per 10‐year increase, 1.21; 95% CI, 0.02 to 2.42; *p* = 0.047) (Figure 2A). Interpreted on the MD scale, each 10‐year increase in mean age corresponded to an approximately 1.21‐point smaller improvement between treatment and control. Including age as a moderator reduced between‐trial heterogeneity ($\tau_{\text{residual}}^{2}$ = 0.74), with 25.4% of heterogeneity explained ($R^{2}$) (Table 2).

**TABLE 2:** Meta‐regression results.

Outcome	Subset	*k*	Pooled MD (RE)	95% CI (RE)	*τ* ^2^ (RE)	*I* ^2^ (RE, %)	*β* per 10y	95% CI (*β*)	*p* (*β*)	*τ* ^2^ residual	*I* ^2^ residual (%)	*R* ^2^ explained (%)
QMG	All studies	18	−2.40	−3.10, −1.71	0.998	60.7	1.21	0.02, 2.41	0.0474	0.744	53.2	25.4
QMG	FCRN only	7	−3.01	−5.10, −0.92	3.918	81.1	3.27	0.87, 5.66	0.0171	0.919	54.3	76.6
QMG	C5IT only	4	−2.40	−3.09, −1.71	0.000	0.0	0.38	−2.96, 3.72	0.6747	0.000	0.0	0.0
QMG	*B*‐cell targeted only	4	−2.12	−3.20, −1.04	0.000	0.0	0.36	−2.57, 3.30	0.6465	0.000	0.0	0.0
MG‐ADL	All studies	18	−1.59	−1.87, −1.31	0.000	0.0	0.08	−0.50, 0.66	0.7785	0.021	0.0	0.0
MG‐ADL	FCRN only	7	−1.85	−2.32, −1.39	0.000	0.0	0.33	−0.76, 1.43	0.4722	0.030	0.0	0.0
MG‐ADL	C5IT only	4	−1.82	−2.42, −1.21	0.000	0.0	−0.23	−3.19, 2.74	0.7704	0.000	0.0	0.0
MG‐ADL	*B*‐cell targeted only	4	−1.55	−2.38, −0.72	0.000	0.0	0.49	−1.18, 2.15	0.3353	0.000	0.0	0.0

*Note:* Analyses were conducted separately for the QMG and MG‐ADL scores and within treatment classes. The *β* is reported per 10‐year increase in mean trial age.

Abbreviations: *β* per 10y, change in MD per 10‐year increase in mean study age; *I* ^2^, proportion of variability due to heterogeneity; MD, mean difference (treatment—placebo) in change from baseline; *R* ^2^, proportion of heterogeneity explained by the moderator; RE, random effects; *τ* ^2^, between‐study variance.

In treatment‐class subgroup meta‐regressions for the QMG, age association was driven by FcRn inhibitor trials (*k* = 7; *β* per 10 years, 3.92; 95% CI, 0.87 to 5.66), rather than C5IT trials (*k* = 4; *β* per 10 years, 0.38; 95% CI, −2.96 to 3.72) or *B* cell‐targeted therapies (*k* = 4; *β* per 10 years, 0.36; 95% CI, −2.58 to 3.30), noting the limited number of studies within each class (Figure 2B–D).

#### 3.4.2 Mg‐ADL

**FIGURE 3:**
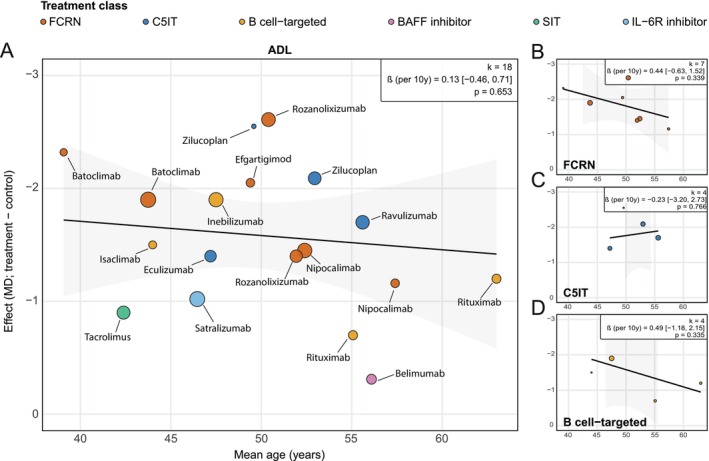
Association of Age with the Treatment Effect on the MG‐ADL score. (A) Bubble plot of trial‐specific mean differences (treatment–control) in change from baseline for the activities of daily living (ADL) score for each, plotted against mean baseline age. Bubble size is proportional to the inverse‐variance weight in the random‐effects model. The solid line indicates the fitted meta‐regression; shading indicates the 95% confidence interval (CI) around the fitted line. The *y*‐axis is inverted so that more negative values (greater improvement with treatment) appear higher. (B) Meta‐regression restricted to FcRn inhibitor trials. (C) Meta‐regression restricted to complement inhibitor (C5IT) trials. (D) Meta‐regression restricted to B cell‐targeting trials. Treatment classes are indicated by point color. ADL, activities of daily living; BAFF, *B*‐cell activating factor; CI, confidence interval; C5IT, complement C5 inhibitor therapy; CD20‐mAb, anti‐CD20 monoclonal antibody; FcRn, neonatal Fc receptor; MD, mean difference; MG, myasthenia gravis; nCD40 mAb, anti‐CD40 monoclonal antibody; SIT, standard immunosuppressive therapy.

In contrast, trial‐level mean age was not associated with the treatment effect on the MG‐ADL scale (*β* per 10‐year increase, 0.08; 95% CI, −0.50 to 0.66; *p* = 0.779) (Figure 3A). Consistent with this, residual heterogeneity remained negligible after accounting for age ($\tau_{\text{residual}}^{2}$ = 0.000; $R^{2}$ explained 0.0%).

Treatment‐class subgroup analyses did not suggest age effect modification of the MG‐ADL in either FcRn inhibitor (*k* = 7; *β* per 10 years, 0.33; 95% CI, −0.76 to 1.43), C5IT (*k* = 4; *β* per 10 years, −0.23; 95% CI, −3.19 to 2.74) or *B* cell‐targeted trials (*k* = 4; *β* per 10 years, 0.49; 95% CI, −1.18 to 2.16), again with limited studies per class (Figure 3B–D).

### 3.5 Sensitivity Analysis

Sensitivity analyses were consistent with the primary meta‐regression findings (Table S2). For QMG, the age‐associated attenuation of treatment effect was unchanged after excluding median‐imputed inputs (*β* per 10 years, 1.38; 95% CI, 0.17 to 2.59; *p* = 0.028) and remained directionally similar when restricting to estimates derived from reported contrasts or CIs, although with wider uncertainty (*k* = 13; *β* per 10 years, 1.19; 95% CI, 0.43 to 2.81; *p* = 0.045). Restricting to trials in the targeted classes yielded a larger age slope (*k* = 11; *β* per 10 years, 1.82; 95% CI, 0.45 to 3.19; *p* = 0.013), and age continued to account for a meaningful proportion of residual heterogeneity across sensitivity sets (residual $\tau^{2}$ range, 0.57–0.74; $R^{2}$ explained, 44%–56%). In contrast, for the MG‐ADL scale, age was not associated with treatment effects in any sensitivity set (*β* per 10 years range, −0.17 to 0.30; all *p* ≥ 0.22), with negligible residual heterogeneity. In the sensitivity analyses, the association between higher trial‐level age and smaller QMG treatment effects remained directionally consistent and was generally robust to alternative analytic restrictions, whereas no evidence of age‐related effect modification emerged for MG‐ADL under any scenario. Together, these findings support the stability of the primary meta‐regression analysis.

## 4 Discussion

Across randomized clinical trials of MG therapies, pooled effects favored treatment on both assessed scales. Meta‐regression identified baseline age as a meaningful source of between‐trial variability for the QMG score: trials enrolling older populations showed smaller benefits for the QMG score, and including age explained a substantial fraction of heterogeneity. In contrast, effects for the MG‐ADL score showed no evidence of age‐related modification of efficacy. These results provide evidence that age is clinically relevant for interpreting treatment effects of MG therapies.

The observed age association may reflect, at least in part, biological differences in treatment responsiveness. Aging is accompanied by immunosenescence and a greater burden of comorbidity, both of which may influence immune function and pharmacologic vulnerability [18]. In parallel, late‐onset MG appears to represent a distinct immunobiology, as supported by an altered serum proteome [19] and immune‐cell patterns consistent with enhanced immunosenescence [15]. In class‐specific meta‐regressions for QMG, the age‐associated attenuation of treatment benefit was stronger in FcRn inhibitor trials (*β*, 3.92 points per 10 years) than in C5 inhibitor (*β*, 0.38 points per 10 years) or *B* cell–targeted trials (*β*, 0.36 points per 10 years). Although exploratory given the limited number of trials within each subclass, this pattern raises the possibility that age‐related biological differences may be particularly relevant to FcRn‐directed therapy. FcRn is a central regulator of IgG salvage and trafficking. Experimental studies have shown that IgG accumulates with age, particularly in white adipose tissue, and that this process depends on FcRn‐mediated recycling, supporting the concept that the IgG‐FcRn axis is altered in aging [20]. Aging has also been associated with changes in IgG glycosylation that may shift the inflammatory properties of IgG and modify downstream Fc‐mediated immune signaling [21]. Further support for biological differences between early‐ and late‐onset MG comes from genetic studies. A recent genome‐wide meta‐analysis identified distinct risk loci in early‐ and late‐onset MG [22]. These observations support that age‐related changes in IgG handling may influence apparent treatment responsiveness. At the same time, trial‐level meta‐regression cannot disentangle such mechanisms from age‐correlated differences in trial design or population characteristics.

A second, complementary explanation is the divergence between impairment‐based and function‐based endpoints. The QMG is an examiner‐dependent impairment scale assessed at a single visit, whereas the MG‐ADL is a patient‐reported measure of day‐to‐day function over a defined recall period [23, 24]. The finding that age was associated with attenuation of treatment effects on QMG but not MG‐ADL may therefore reflect differences in what these instruments capture across clinical contexts [25, 26]. Older trial populations may have a greater burden of non–MG‐related limitations, such as sarcopenia, frailty, cardiovascular disease or neuropathies, that can constrain performance on timed or strength tasks and reduce the apparent magnitude of change on impairment scales even when MG‐specific symptoms improve [27]. Differences in therapeutic dynamics and endpoint timing may further accentuate this pattern. FcRn inhibitors are often evaluated in cycle‐based regimens with primary efficacy assessed near the period of maximal clinical response, when effects may peak and subsequently wane [4, 6]. By contrast, trials of C5 inhibitors and *B* cell–targeted therapies were typically designed with fixed dosing intervals and assessed clinical outcomes at predefined time points (such as week 26 for eculizumab [9] or ravulizumab [28]). This explanation, however, may not fully account for the observed pattern, as nipocalimab produces sustained IgG reduction over 24 weeks [8], yet showed a directionally similar age‐associated pattern in this meta‐analysis. Taken together, the endpoint‐specific pattern observed here likely reflects a combination of biological and measurement‐related factors that warrants further study.

These findings have practical implications for the design and interpretation of future MG trials. Future studies should be mindful of upper age limits, ensure adequate representation of older patients, and prespecify age as a stratification factor or covariate in the statistical analysis plan. In addition, structured assessment of comorbidity, frailty, baseline physical reserve, and concomitant medication burden may help distinguish true age‐related differences in treatment responsiveness from age‐related differences in endpoint performance. Given the rarity of MG, separate trials for older and younger patients are unlikely to be feasible in the near term. Instead, real‐world data may provide the most practical route toward age‐informed MG therapeutics. This data could be used to assess whether age modifies treatment effectiveness, safety, and treatment discontinuation in routine care in the context of aging.

### 4.1 Strengths and Limitations

This study leveraged the recent expansion of randomized clinical trials in MG, enabled by the advent of mechanism‐targeted therapies, to synthesize evidence from 18 trials including 1823 participants. Use of a preregistered protocol and PRISMA‐concordant methods with prespecified analytic decisions strengthened internal consistency, and the primary inferences were corroborated across sensitivity analyses.

However, several limitations warrant consideration. Because age was available only as a trial‐level mean, the meta‐regression estimates an association between mean age and treatment effect rather than the within‐trial patient‐level interaction; such associations are observational and can be distorted by ecological (aggregation) bias and confounding from systematic differences between trials [29]. Accordingly, the observed age slope may partly reflect age‐linked differences in trial populations and design rather than age itself acting as a biological effect modifier. Further, subgroup‐specific analyses were based on fewer trials and were particularly susceptible to confounding. These subgroups should therefore be interpreted with caution. Finally, primary efficacy time points differed across trials and mechanisms, which may have contributed to between‐study heterogeneity and may partly confound the observed age associations.

## 5 Conclusion

Across randomized clinical trials of MG therapies, treatment was associated with clinical improvements, but higher trial‐level age was associated with attenuation of treatment effects on the QMG and not on the MG‐ADL scale. These findings suggest that age may contribute to differences in observed treatment effects across MG trials and support patient‐level analyses to determine whether age modifies treatment response and, if so, through which biological or clinical pathways.

## Author Contributions

**Christopher Nelke:** conceptualization, methodology, investigation, writing – original draft, writing – review and editing. **Tobias Ruck:** conceptualization, writing – review and editing, writing – original draft, funding acquisition. **Lukas Theissen:** writing – review and editing, validation. **Francesco Sacca:** validation, writing – review and editing. **Marion Skrobol:** writing – review and editing, investigation, formal analysis, data curation.

## Funding

This work was funded by the Else Kröner‐Fresenius‐Stiftung to CN (2023_EKEA.38 and 2025 EKMS.O2). Further, this work was supported by the internal research funding program of the Heinrich Heine University to CN. Further study funding by the DGM (Deutsche Gesellschaft für Muskelkranke) to TR and to CN, by the German Research Foundation (DFG, to CN: NE 2774/2‐1, to TR: 549557400/RU 2169/5‐1, 417677437/GRK2578), by the EFRE/JTF program (B2B‐RARE, to TR: EFRE‐20800340) and by the InnovationsFoRUM of the RUB—Regulation of IIM (IF‐045‐25).

## Conflicts of Interest

The authors declare no conflicts of interest.

## Supporting information


**Figure S1:** PRISMA Flow Diagram of Study Identification, Screening, and Inclusion. Flow diagram summarizing the identification of records and the inclusion of randomized clinical trials in the systematic review and meta‐analysis.

**Figure S2:** Risk of Bias Assessment for Included Randomized Clinical Trials. Risk of bias for each included trial was assessed using the Cochrane Risk of Bias 2 tool across the indicated domains. Colors denote judgments of low risk of bias (green) or some concerns (yellow). There were no studies with high risk of bias in any domain. The overall risk of bias judgment is shown in the final column.

**Table S1:** Eligibility Criteria for Study Inclusion and Exclusion. Eligibility criteria were defined before study selection and were structured according to the PICO framework.

**Table S2:** Sensitivity analyses for age meta‐regression. Values are *β* per 10‐year increase in mean trial age (95% CI) from random‐effects meta‐regression models, shown separately for QMG and MG‐ADL. *β* per 10y, change in MD per 10‐year increase in mean study age; *τ* ^2^, between‐study variance; *I* ^2^, proportion of variability due to heterogeneity; *R* ^2^, proportion of heterogeneity explained by the moderator; RE, random effects; MD, mean difference (treatment—control) in change from baseline.

## Data Availability

The data that supports the findings of this study are available in the Supporting Information of this article.
