# Supplementary material for: Age and Treatment Efficacy in Myasthenia Gravis: A Systematic Review, Meta‐Analysis, and Meta‐Regression
Source: Eur J Neurol. 2026 Aug 12;33(8):e70737. doi: 10.1111/ene.70737 (PMC13469751; doi:10.1111/ene.70737)
Supplement: Supplementary file 1 [file ENE-33-e70737-s001.pdf]

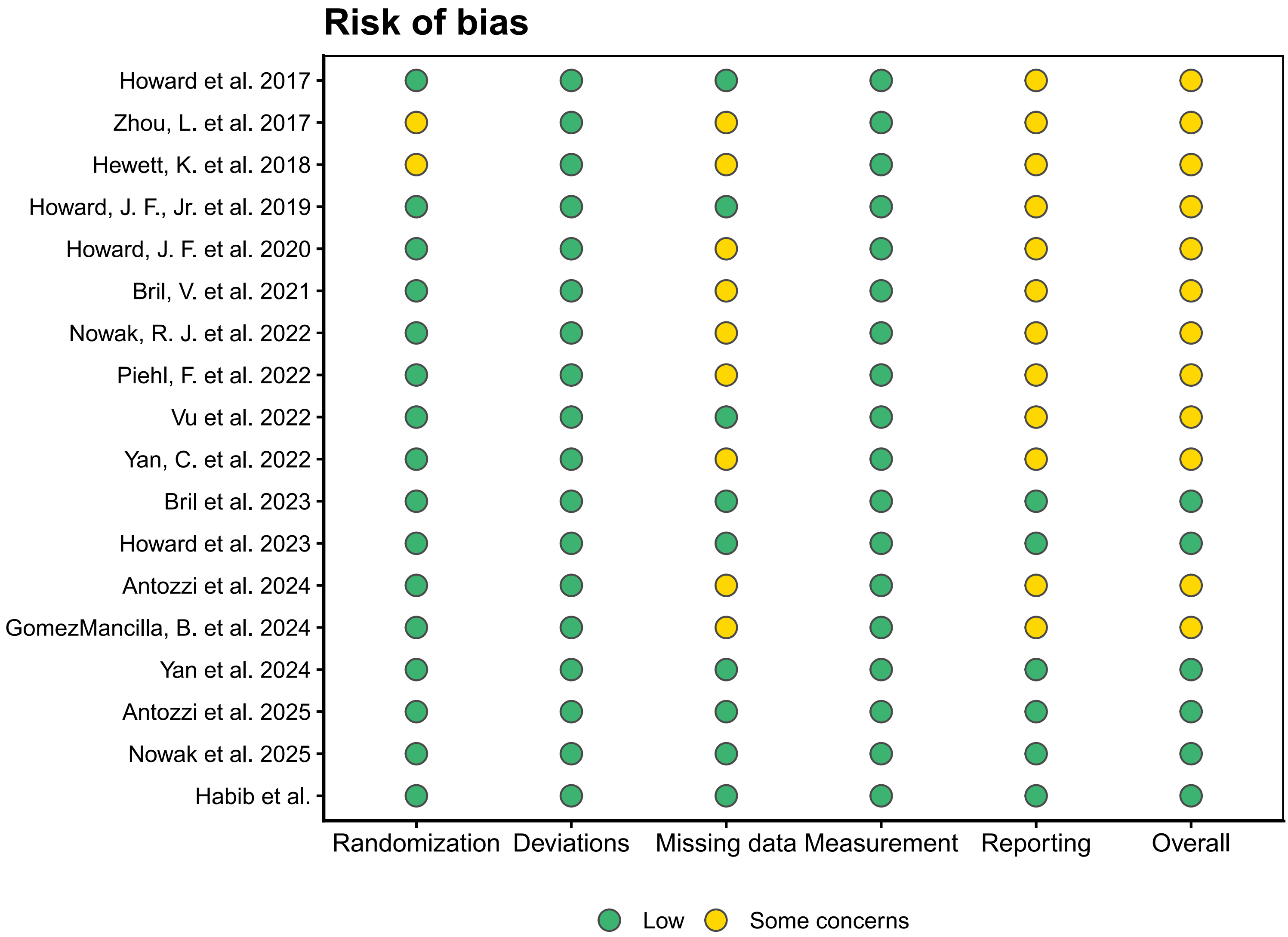
Risk of bias
Howard et al. 2017
Zhou, L. et al. 2017
Hewett, K. et al. 2018
Howard, J. F., Jr. et al. 2019
Howard, J. F. et al. 2020
Bril, V. et al. 2021
Nowak, R. J. et al. 2022
Piehl, F. et al. 2022
Vu et al. 2022
Yan, C. et al. 2022
Bril et al. 2023
Howard et al. 2023
Antozzi et al. 2024
GomezMancilla, B. et al. 2024
Yan et al. 2024
Antozzi et al. 2025
Nowak et al. 2025
Habib et al.
Randomization
Deviations
Missing data
Measurement
Reporting
Overall
Low
Some concerns
