# Supplementary material for: Age and Treatment Efficacy in Myasthenia Gravis: A Systematic Review, Meta‐Analysis, and Meta‐Regression
Source: Eur J Neurol. 2026 Aug 12;33(8):e70737. doi: 10.1111/ene.70737 (PMC13469751; doi:10.1111/ene.70737)
Supplement: Supplementary file 2 [file ENE-33-e70737-s002.pdf]

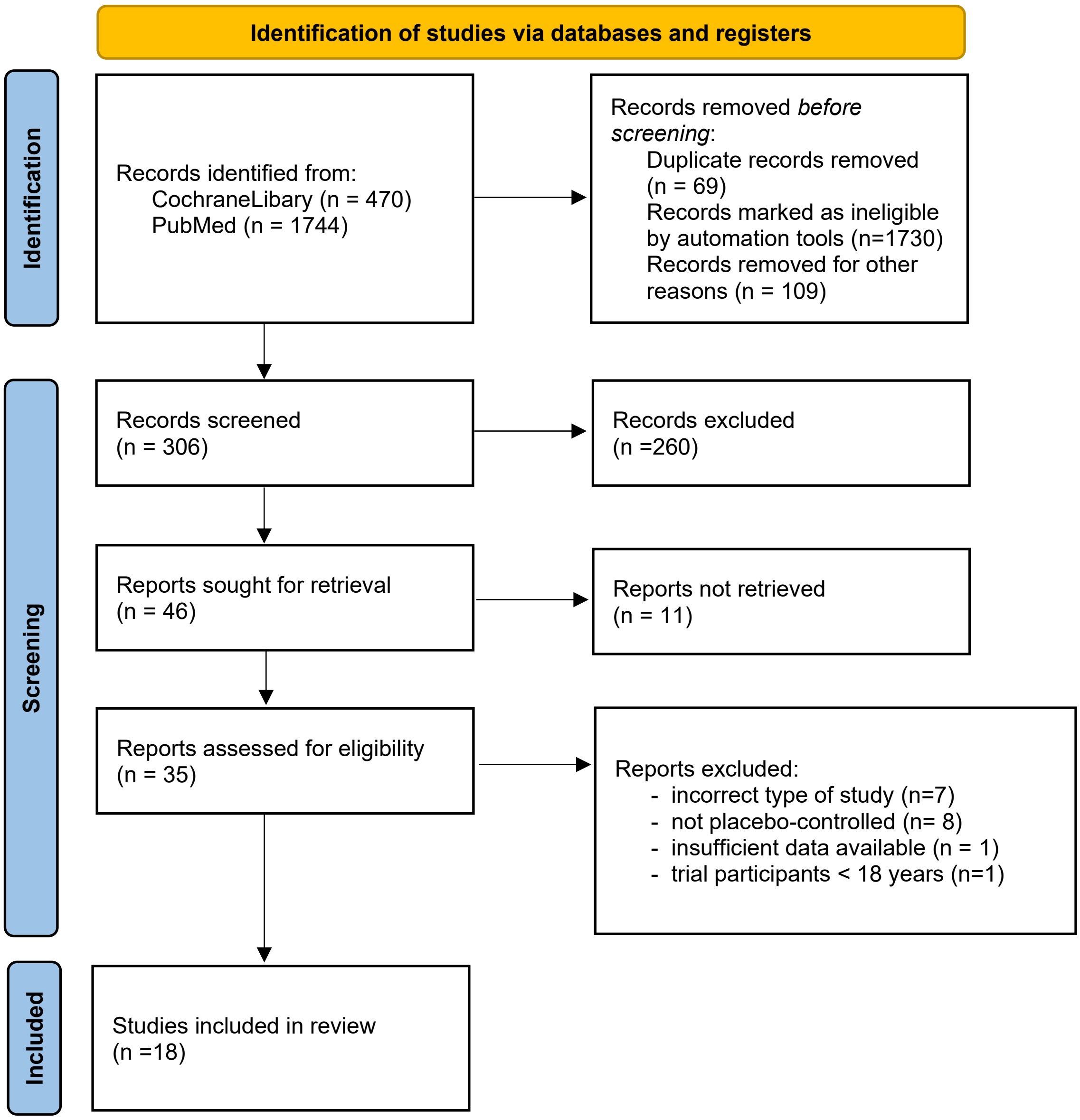
Identification of studies via databases and registers
Identification
Records identified from:
CochraneLibary (n = 470)
PubMed (n = 1744)
Records removed *before screening*:
Duplicate records removed (n = 69)
Records marked as ineligible by automation tools (n=1730)
Records removed for other reasons (n = 109)
Screening
Records screened (n = 306)
Records excluded (n =260)
Reports sought for retrieval (n = 46)
Reports not retrieved (n = 11)
Reports assessed for eligibility (n = 35)
Reports excluded:
- incorrect type of study (n=7)
- not placebo-controlled (n= 8)
- insufficient data available (n = 1)
- trial participants < 18 years (n=1)
Included
Studies included in review (n =18)
