# Supplementary material for: Age and Treatment Efficacy in Myasthenia Gravis: A Systematic Review, Meta‐Analysis, and Meta‐Regression
Source: Eur J Neurol. 2026 Aug 12;33(8):e70737. doi: 10.1111/ene.70737 (PMC13469751; doi:10.1111/ene.70737)
Supplement: Supplementary file 3 [file ENE-33-e70737-s003.docx]

**Supplementary Table 1. Eligibility Criteria for Study Inclusion and Exclusion**

Eligibility criteria were defined before study selection and were structured according to the PICO framework.

| Domain | Inclusion criteria | Exclusion criteria |
| --- | --- | --- |
| Population | Adults with myasthenia gravis. Studies enrolling generalized MG populations were eligible. | Paediatric-only populations; studies not focused on MG; mixed neuromuscular cohorts if MG-specific data could not be extracted. |
| Disease definition | Studies in which MG was clinically diagnosed according to trial-defined criteria. | Studies without a clear MG diagnosis or without extractable data for patients with MG. |
| Intervention | Pharmacologic disease-modifying or immunomodulatory treatment for MG, including targeted therapies and conventional immunosuppressive treatment. | Symptomatic-only interventions, non-pharmacologic interventions, surgical interventions, diagnostic studies, or studies without an active therapeutic intervention. |
| Comparator | Placebo, standard care, or control arm allowing estimation of a between-group treatment effect. | Single-arm studies; uncontrolled extension studies; studies without a concurrent comparator. |
| Study design | Randomized controlled clinical trials, including phase 2 and phase 3 trials. | Observational cohort studies, case-control studies, case series, case reports, open-label extension studies without randomized comparison, reviews, editorials, protocols, conference abstracts without sufficient outcome data, and preclinical studies. |
| Outcomes | Reported sufficient data to estimate treatment effects for at least one prespecified efficacy outcome: QMG and/or MG-ADL change from baseline at the trial’s prespecified primary efficacy time point or a clinically meaningful efficacy time point. | Studies not reporting QMG or MG-ADL outcomes, or reporting outcomes in a form from which a treatment effect and corresponding uncertainty could not be extracted or derived. |
| Age data | Studies reporting trial-level baseline age, preferably as mean age. When median age was reported, conversion or approximation was considered. | Studies without any extractable baseline age information for the analysed trial population. |
| Publication type | Full-text peer-reviewed publications and complete trial reports with sufficient methodological and numerical detail. | Abstract-only reports, narrative reviews, systematic reviews, meta-analyses, letters without original trial data, and secondary analyses that duplicated a primary trial report unless they provided otherwise unavailable numerical data. |
| Language | Publications in English. | Non-English publications for which the relevant trial data could not be reliably extracted. |
| Time frame | Trials published from database inception through the final search date. | Publications outside the search window. |
| Duplicate reports | When multiple publications reported the same trial, the most complete report was used; supplementary or secondary reports were used only to obtain missing eligible data. | Duplicate publications were excluded from the primary dataset to avoid double-counting participants. |
