# Supplementary material for: Age and Treatment Efficacy in Myasthenia Gravis: A Systematic Review, Meta‐Analysis, and Meta‐Regression
Source: Eur J Neurol. 2026 Aug 12;33(8):e70737. doi: 10.1111/ene.70737 (PMC13469751; doi:10.1111/ene.70737)
Supplement: Supplementary file 4 [file ENE-33-e70737-s004.docx]

**Supplemental Table 2: Sensitivity analyses for age meta-regression**

Values are β per 10-year increase in mean trial age (95% CI) from random-effects meta-regression models, shown separately for QMG and MG-ADL.

| **Sensitivity set** | **k (QMG)** | **k (MG-ADL)** | **β per 10y (QMG)** | **β per 10y (MG-ADL)** | **P (QMG)** | **P (MG-ADL)** | **τ² residual (QMG)** | **τ² residual (MG-ADL)** | **I² residual (QMG)** | **I² residual (MG-ADL)** | **R² explained (QMG)** | **R² explained (MG-ADL)** |
| --- | --- | --- | --- | --- | --- | --- | --- | --- | --- | --- | --- | --- |
| Primary (all studies) | 18 | 18 | 1.21 (0.02, 2.41) | 0.08 (-0.50, 0.66) | 0.0474 | 0.7785 | 0.7440 | 0.0210 | 53.2% | 0.0% | 25.4% | 0.0% |
| Exclude median-imputed inputs | 17 | 17 | 1.38 (0.17, 2.59) | 0.09 (-0.52, 0.70) | 0.0284 | 0.7599 | 0.7230 | 0.0290 | 53.1% | 0.0% | 29.9% | 0.0% |
| Contrast/CI-derived only | 13 | 11 | 1.19 (-0.43, 2.81) | -0.17 (-1.16, 0.82) | 0.0455 | 0.7042 | 0.7730 | 0.0400 | 56.0% | 0.0% | 0.0% | 0.0% |
| Targeted classes only | 15 | 15 | 1.82 (0.45, 3.19) | 0.30 (-0.22, 0.82) | 0.0130 | 0.2288 | 0.5710 | 0.0000 | 44.0% | 0.0% | 56.8% | 0.0% |

**Abbreviations**: MD = mean difference (treatment − control) in change from baseline; RE = random effects; β per 10y = change in MD per 10-year increase in mean study age; τ² = between-study variance; I² = proportion of variability due to heterogeneity; R² = proportion of heterogeneity explained by the moderator.
